# MicroRNA-372 inhibits endometrial carcinoma development by targeting the expression of the Ras homolog gene family member C (RhoC)

**DOI:** 10.18632/oncotarget.6544

**Published:** 2015-12-09

**Authors:** Bo-Liang Liu, Kai-Xuan Sun, Zhi-Hong Zong, Shuo Chen, Yang Zhao

**Affiliations:** ^1^ Department of Gynecology, The First Affiliated Hospital of China Medical University, Shenyang 110001, China; ^2^ Department of Biochemistry and Molecular Biology, College of Basic Medicine, China Medical University, Shenyang 100013, China

**Keywords:** endometrial carcinoma, microRNA-372, RhoC, tumorigenesis, progression

## Abstract

Here we explore the role of microRNA-372 (miR-372) in tumorigenesis and development of endometrial adenocarcinoma (EC) and analyze the underlying mechanism. We found that miR-372 expression is much lower in EC than normal endometrial specimens. Cell function experiments demonstrated that miR-372 overexpression suppressed cell proliferation, migration, and invasion, and led to a G1 phase arrest and promoted the apoptosis of endometrial carcinoma cells *in vitro*. The nude mouse xenograft assay demonstrated that miR-372 overexpression suppressed tumor growth. RT-PCR and Western blot assays detected the expression of known targets of miR-372 in other malignant tumors and found Cyclin A1 and Cyclin-dependent Kinase 2 (CDK2) was downregulated by miR-372. Bioinformatic predictions and dual-luciferase reporter assays found that RhoC was a possible target of miR-372. RT-PCR and Western blot assays demonstrated that miR-372 transfection reduced the expression of RhoC, matrix metalloproteinase 2 (MMP2) and MMP9, while it increased the expression of cleaved poly (ADP ribose) polymerase (PARP) and bcl-2-associated X protein (Bax). The cell function experiments that transfected siRNA with RhoC showed the same trend as those which were transfected with miR-372. Taken together, our results demonstrated for the first time that miR-372 suppresses tumorigenesis and the development of EC; RhoC is a new and potentially important therapeutic target.

## INTRODUCTION

Endometrial cancers are one of the most common gynecological cancers in many developed countries [1]. In 2015, the American Cancer Society (ACS) estimates that 54,870 women will be diagnosed with endometrial cancer and 10,170 women will die from this disease [2]. Therefore, it is essential to dissect the underlying molecular mechanisms of tumorigenesis and development of EC for better diagnosis and treatment.

MicroRNAs (miRNAs), a class of small non-coding RNAs (21–23 nt in length), repress the expression of target genes at the post-transcriptional level by binding to the 3′UTR of their target mRNAs to induce degradation or restrain translation [3–4]. MiRNAs have important functions in the development of cell differentiation and the regulation of the cell cycle and apoptosis [5–9]. In the present study, we focus on miR-372, which has been demonstrated to act as an anti-oncogenic miRNA in both cervical carcinoma and hepatocellular carcinoma, and to act as an oncogenic miRNA in testicular germ cell tumors, human embryonic stem cells (hESCs), head and neck squamous cell carcinoma (HNSCC), and colorectal cancer. However, the role of miR-372 in endometrial cancer remains unclear. Our results show that miR-372 suppresses the tumorigenesis and development of EC.

## RESULTS

### Correlation of miR-372 expression with the pathogenesis and aggressiveness of endometrial carcinoma

**Figure 1:**
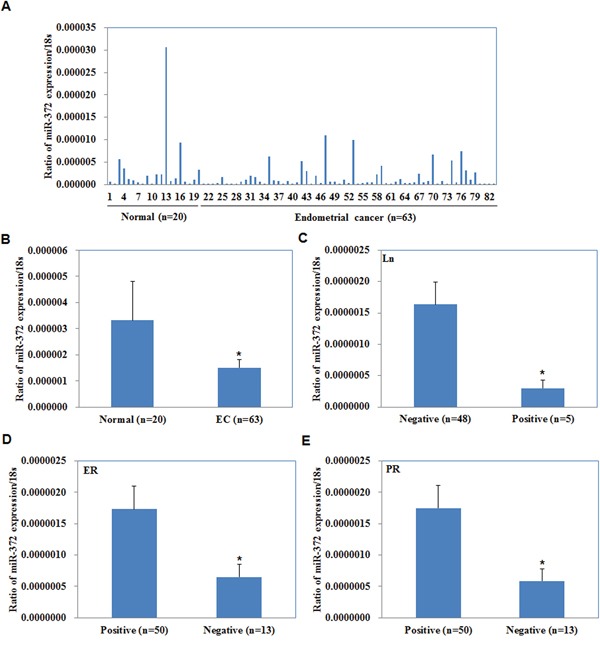
Correlation of miR-372 expression with pathogenesis and aggressiveness of endometrial carcinoma MiR-372 mRNA expression was significantly lower in endometrial adenocarcinoma than in normal endometrial tissues **A/B.** and was negatively associated with Lymph node metastasis **C.** positively associated with ER **D.** and PR expression **E.** EC = endometrial adenocarcinoma, ER = estrogen receptor, PR = progesterone receptor.

The expression levels of miR-372 were analyzed in endometrial carcinoma samples and normal samples by qRT-PCR. As shown in Figure 1A, miR-372 expression levels were lower in EC tissues than those in normal samples (*P* < 0.05, Figure 1B), and in lymph nodes metastasis (+) than those in lymph nodes metastasis (−) (*P* < 0.05, Figure 1C). Besides, miR-372 expression was lower in estrogen receptor (ER) and progesterone receptor (PR) negative tissues than in ER and PR positive tissues (*P* < 0.05, Figure 1D & 1E).

### MiR-372 overexpression suppresses endometrial carcinoma cell proliferation

**Figure 2:**
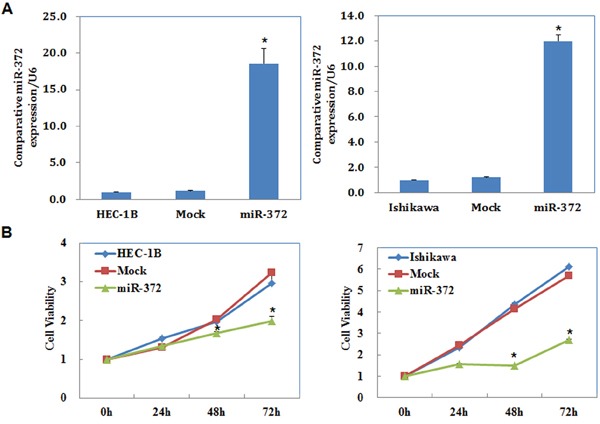
MiR-372 overexpression suppresses endometrial carcinoma cell proliferation *in vitro* Following miR-372 transfection, endometrial adenocarcinoma cell lines exhibited significantly higher miR-372 expression **A.** and slower growth **B.** compared with the control and mock cells. Results are representative of three separate experiments; data are expressed as the mean ± standard deviation, *P < 0.05.

The miR-372 mimics were transfected into cells to upregulate miR-372 expression. We analyzed miR-372 levels after transfection by qRT-PCR and found that miR-372 levels were significantly increased (*P* < 0.05; Figure 2A).

We performed a MTT proliferation assay after miR-372 transfection. A significant reduction of cell viability was observed 48, and 72 hours after transfection with the miR-372 mimics compared with control and mock-transfected cells (*P* < 0.05; Figure 2B).

### MiR-372 overexpression induces G1 phase arrest and promotes apoptosis of endometrial carcinoma cells

**Figure 3:**
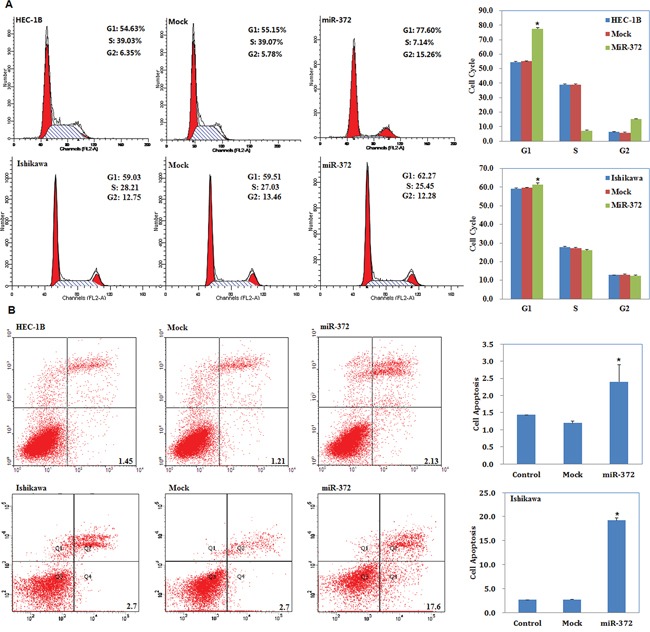
MiR-372 overexpression induces G1 phase arrest and promotes apoptosis of endometrial carcinoma cells Following miR-372 transfection, endometrial adenocarcinoma cell lines exhibited significantly induced G1 arrest **A.** and elevated apoptosis **B.** compared with the control and mock cells. Results are representative of three separate experiments; data are expressed as the mean ± standard deviation, *P < 0.05.

Cell cycle analysis demonstrated that miR-372 transfection increased the percentage of cells in G1 phase versus control and mock-transfected cells (*P* < 0.05; Figure 3A). Apoptosis assays demonstrated that cell apoptosis rates were elevated 48 hours after transfection with the miR-372 mimics compared with control and mock-transfected cells (*P* < 0.05; Figure 3B).

### MiR-372 overexpression suppresses endometrial carcinoma cell migration and invasion

**Figure 4:**
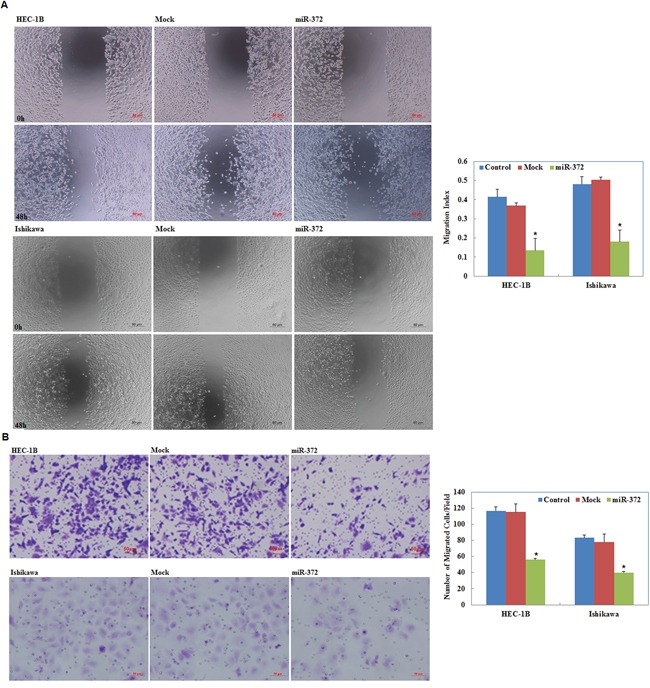
Effects of miR-372 transfection on invasive and metastatic ability of endometrial adenocarcinoma cell lines *in vitro* Following miR-372 transfection, endometrial adenocarcinoma cell lines exhibited lower migration in wound healing assays **A.** and slower invasion in Transwell assays **B.** compared with the control and mock cells. Results are representative of three separate experiments; data are expressed as the mean ± standard deviation, *P < 0.05.

Our wound-healing assay showed that cells overexpressing miR-372 presented a slower closing of the scratch wound compared with the control and mock-transfected cells (*P* < 0.05; Figure 4A). Transwell assays showed that the cells transfected with miR-372 significantly reduced the ability to invade compared with control and mock-transfected cells (*P* < 0.05; Figure 4B).

### MiR-372 overexpression suppresses the tumorigenicity of endometrial carcinoma cells *in vivo*

**Figure 5:**
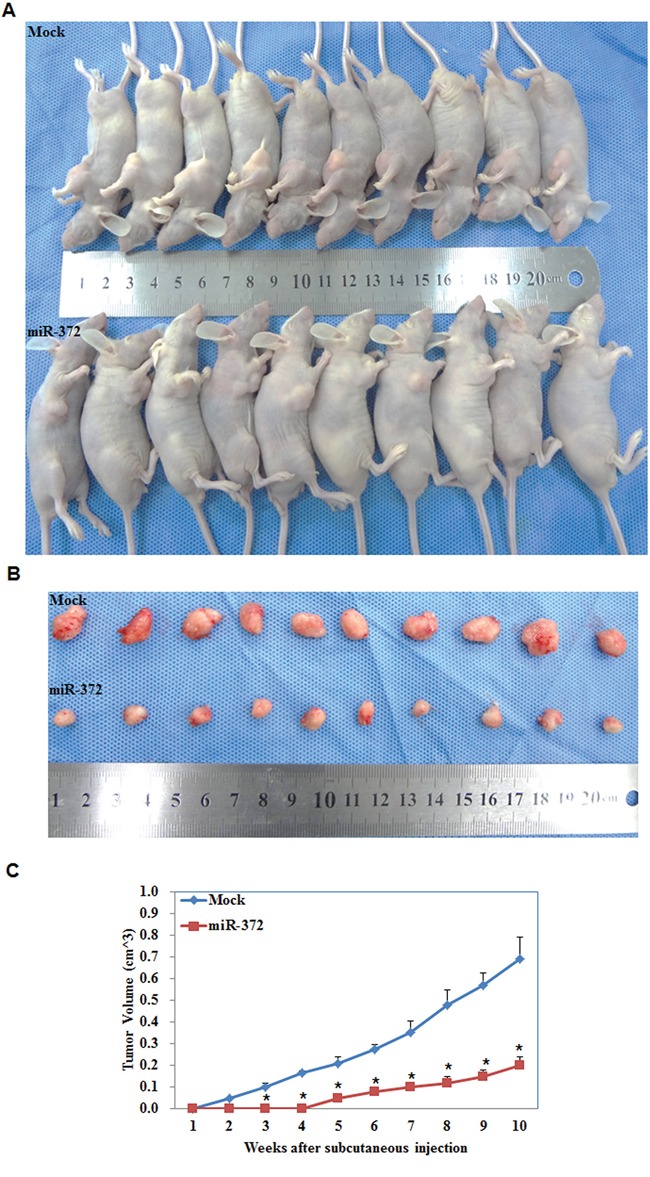
MiR-372 inhibited tumor growth *in vivo* Tumor xenograft volume in nude mice treated with miR-372 was smaller than that in mock nude mice **A & B.** Tumor xenograft growth in miR-372-treated nude mice was slower than that in the control group **C.** *P < 0.05.

We performed nude mice xenograft assays and demonstrated that, compared with the control group, in mice injected with the HSA-372 transfection cells tumorigenicity was slower (Figure 5A & 5B) and, given the same duration of observation, the volume of tumors was smaller (*P* < 0.05; Figure 5C).

### Cyclin A1 and CDK2 were downregulated in endometrial carcinoma cells transfected with miR-372, and in the tumor tissues of the HSA-372 group of nude mice, while ATAD2, Lats2, P21, P62, and DKK1 demonstrated no significant differences in expression

**Figure 6:**
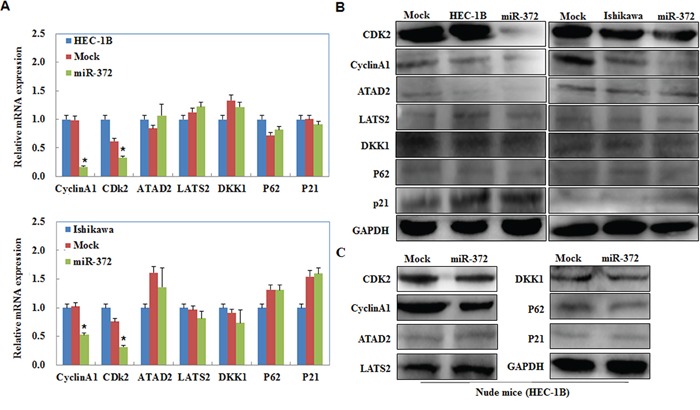
Effects of miR-372 transfection on endometrial adenocarcinoma cell genotype *in vitro* and *in vivo* miR-372 overexpression reduced Cyclin A1, CDK2, however, no significant differences were found in ATAD2, Lats2, P21, P62, and DKK1 expression when compared with mock and control *in vitro* **A & B.** and *in vivo* **C.** *P < 0.05.

We performed Western blot and RT-PCR analysis to detect the expression of Cyclin A1, CDK2, ATAD2, Lats2, P21, P62, and DKK1 in EC cells transfected with miR-372. We showed that Cyclin A1 and CDK2 were downregulated at the protein and mRNA level compared with the negative control. However, ATAD2, Lats2, P21, P62, and DKK1 showed no significant differences in expression level (*P* < 0.05; Figure 6A & 6B). Western blot analysis also demonstrated the same trend, with the expression of Cyclin A1 and CDK2 being downregulated in the tumor tissues of the HSA-372 group of nude mice. Other genes showed no significant differences (*P* < 0.05; Figure 6C).

### RhoC is a target of miR-372

**Figure 7:**
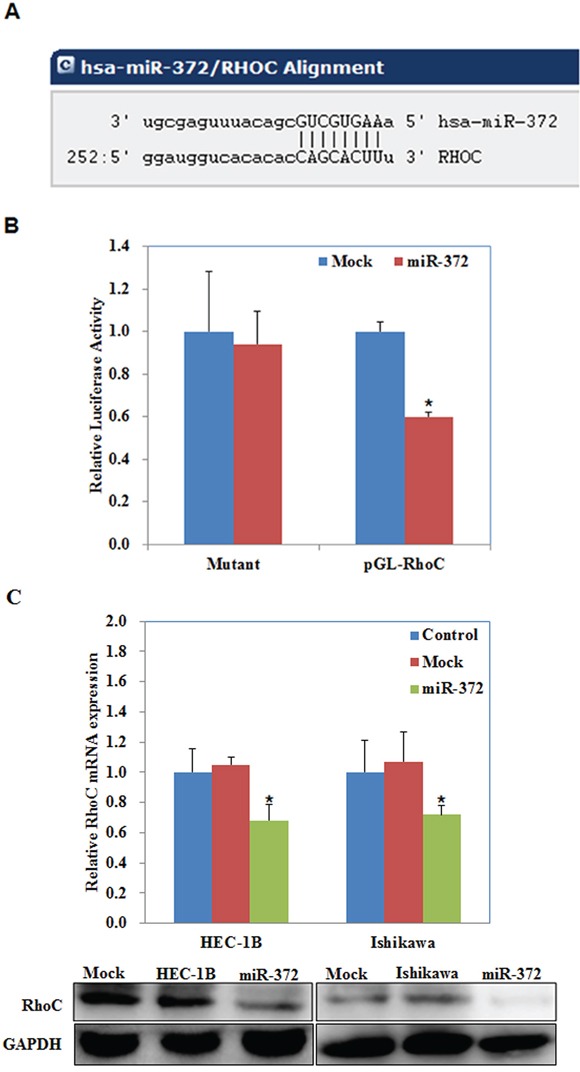
RhoC is a target of miR-372 The predicted seed region in the 3′ UTR of RhoC showed that RhoC was a direct target of miR-372 **A.** dual-luciferase reporter assay indicated that miR-372 directly targeted RhoC by binding its 3′UTR **B.** miR-372 transfection reduced the expression of RhoC at the mRNA and protein levels **C.** *P < 0.05.

**Figure 8:**
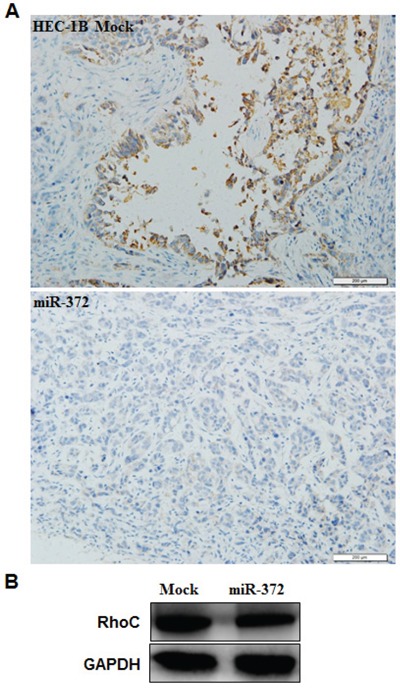
Effects of miR-372 transfection *in vivo* Immunohistochemical and Western blot analysis demonstrated a significant reduction of RhoC expression in the HSA-372 group compared with the control group in nude mice tumor tissues **A & B.** *P < 0.05.

According to a prediction website (http://microRNA.org), we found that the complementary sequence of miR-372 was found in the 3′UTR of RhoC (*P* < 0.05; Figure 7A). We performed luciferase reporter assays with the wild-type or mutant 3′UTR of RhoC. Our results demonstrate that miR-372 significantly decreased the relative luciferase activity of the wild-type RhoC 3′UTR compared with the mutant RhoC 3′UTR, indicating that miR-372 may directly bind to the 3′UTR of RhoC (*P* < 0.05; Figure 7B). QRT-PCR and Western blot analysis showed that the miR-372 transfection reduced the expression of RhoC at both mRNA and protein levels (*P* < 0.05; Figure 7C). Immunohistochemical analysis and Western blot demonstrated a significant reduction of RhoC expression in the HSA-372 group compared with the control group in nude mice tumor tissues (*P* < 0.05; Figure 8A & 8B). Taken together, these results suggest that RhoC is a direct target of miR-372.

### siRhoC transfection suppresses endometrial carcinoma cell proliferation

**Figure 9:**
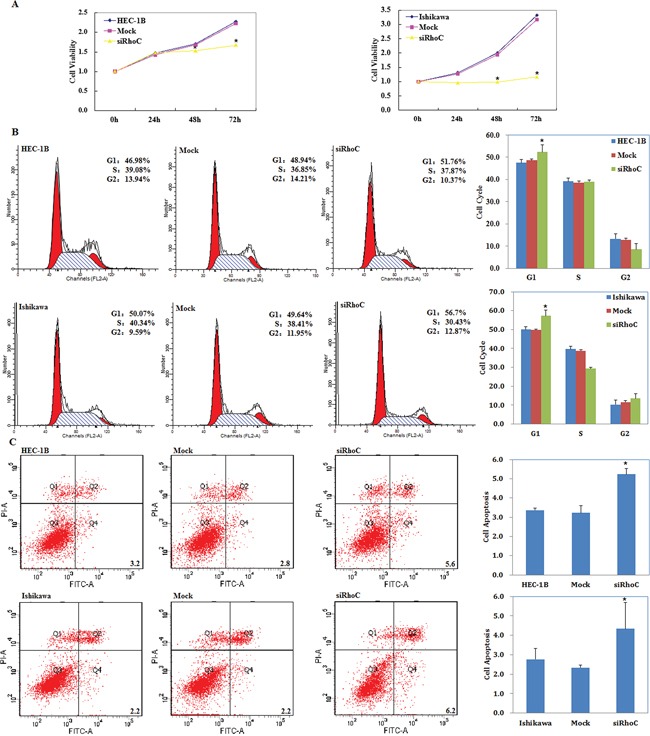
siRhoC suppresses endometrial carcinoma cell proliferation, induces G1 phase arrest and promotes apoptosis of endometrial carcinoma cells Following siRhoC transfection, endometrial adenocarcinoma cell lines exhibited significantly slower growth **A.** G1 arrest **B.** and elevated apoptosis **C.** compared with the control and mock cells. Results are representative of three separate experiments; data are expressed as the mean ± standard deviation, *P < 0.05.

siRhoC was transfected into cells to reduce RhoC expression. We performed a MTT proliferation assay after cells were transfected with siRhoC. A significant reduction of cell viability was observed 48, and 72 hours after transfection with siRhoC compared with control and mock-transfected cells (*P* < 0.05; Figure 9A).

### siRhoC induces G1 phase arrest and promotes apoptosis of endometrial carcinoma cells

Cell cycle analysis demonstrated that after transfection with siRhoC the percentage of cells in G1 phase increased when compared with control and mock-transfected cells (*P* < 0.05; Figure 9B).

Apoptosis assays demonstrated that apoptosis rates were elevated 48 hours after transfection with siRhoC compared with control and mock-transfected cells (*P* < 0.05; Figure 9C).

### siRhoC suppresses endometrial carcinoma cell migration and invasion

**Figure 10:**
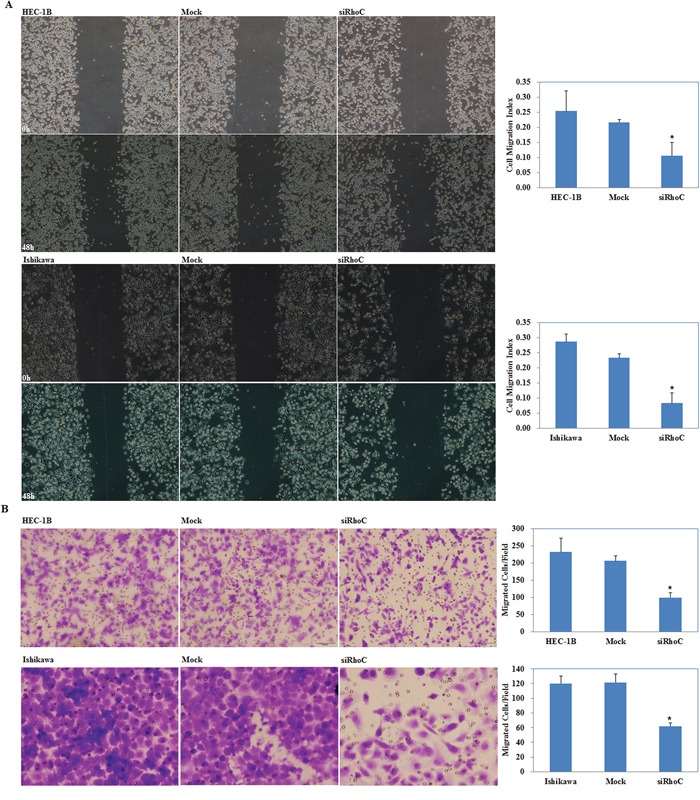
Effects of siRhoC transfection on invasive and metastatic ability of endometrial adenocarcinoma cell lines *in vitro* siRhoC transfection exhibited lower migration in wound healing assays **A.** and slower invasion in transwell assays **B.** compared with the control and mock cells. Results are representative of three separate experiments; data are expressed as the mean ± standard deviation, *P < 0.05.

Our wound-healing assay showed that cells transfected with siRhoC had slower closing of scratch wounds compared with the control and mock-transfected cells (*P* < 0.05; Figure 10A). Transwell assays showed that the cells transfected with siRhoC had significantly reduced ability to invade compared with control and mock-transfected cells. (*P* < 0.05; Figure 10B)

### MiR-372 overexpression regulates MMP2, MMP9, PARP, and BAX mRNA or protein expression

**Figure 11:**
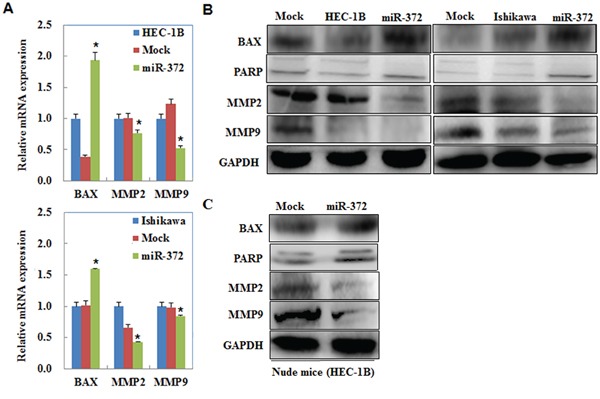
MiR-372 overexpression regulates MMP2, MMP9, PARP, and BAX mRNA or protein expression QRT-PCR and Western blot results showed that mRNA or protein expression of MMP2 and MMP9 were decreased but the expression of cleaved PARP and Bax were increased by miR-372 compared with the negative controls **A & B.** Western blot analysis demonstrated the same trend in the tumor tissues of the HSA-372 group of nude mice **C.**

We performed qRT-PCR and Western blot to measured MMP2, MMP9, PARP, and Bax mRNA or protein expression levels after transfection with the miR-372 mimics in EC cells. mRNA or protein expression of MMP2 and MMP9 were decreased but the expression of cleaved PARP and Bax were increased by miR-372 compared with the negative controls (*P* < 0.05; Figure 11A & 11B). Western blot analysis demonstrated the same trend in the tumor tissues of the HSA-372 group of nude mice (*P* < 0.05; Figure 11C).

## DISCUSSION

We found that the mRNA levels of miR-372 in EC tissue were lower than in normal tissues and lower in lymph nodes with metastases than in lymph nodes without metastases. In addition, miR-372 expression was lower in ER negative and PR negative tissue than ER positive and PR positive tissue. Patients who are ER and PR negative have significantly poorer disease-free survival than those who are ER and PR positive [10], as do those with lymph node metastases. Therefore, miR-372 may be an anti-oncogene in EC. This led us to transfect miR-372 into EC cells to investigate the influence of miR-372 in EC cell tumorigenesis and tumor progression.

Our results demonstrated that miR-372 suppressed the proliferation, migration, and invasion of cells, increased the percentage of cells in G1 phase and promoted the apoptosis of endometrial carcinoma cells. Lastly, we performed nude mice xenograft assays, and the results showed that miR-372 suppressed tumorigenesis and tumor growth. These results all demonstrate an anti-oncogene role of miR-372 in endometrial carcinoma, which is consistent with the role of miR-372 in cervical carcinoma and hepatic carcinogenesis but is contrary to the role of miR-372 in testicular germ cell tumors, hESCs, HNSCC, and colorectal cancer.

MiR-372 suppresses the growth of cervical cancer cells by downregulating cyclin A1 and CDK2 [11]. MiR-372 plays an anti-oncogenetic role through the downregulation of ATAD2 expression in hepatic carcinogenesis [12]. MiR-372 can possibly directly inhibit the expression of the tumor-suppressor Lats2 to promote the development of human testicular germ cell tumors [13]. In a previous study, it was shown that miR-372 could target p21, a tumor suppressor gene, to influence the division of hESCs [14]. MiR-372 decreases p62, thus increasing the motility of HNSCC cells *in vitro* and *in vivo* [15]. A previous study has shown that miR-372 promotes tumor cell proliferation and invasion by modulating DKK1 levels in colorectal cancer [16]. Because microRNA plays a role in tumorigenesis by downregulating its target genes at the post-transcriptional level, we examined the expression levels of Cyclin A1/CDK2, ATAD2, Lats2, P21, P62, and DKK1 in EC cells transfected with miR-372 by qRT-PCR and Western blot. Our results demonstrated that Cyclin A1 and CDK2 mRNA and protein levels decreased compared with the negative control; however, ATAD2, Lats2, P21, P62, and DKK1 showed no significant difference at the mRNA and protein level. Therefore, we determined the expression of them in the tumor tissue from the HSA-372 group of nude mice and found the results to be consistent. The cyclin A1-CDK2 complex is crucial for successful DNA replication and normal cell cycle progression [11]. CDK2 is thought to be essential in the mammalian cell cycle and functions by driving cells through S phase in conjunction with A-type cyclins [17]. CDK2 is expressed at a higher level at the invasive front of EC than it is more deeply within the tumor EC [18]. CDK2 overexpression may be involved in the development and/or progression of human EC [19]. CDK2 expression gradually increases from normal through hyperplasia to carcinoma, indicating its potential importance in both early and late carcinogenesis in EC [20]. Cyclins are regarded as the major regulators of the cell cycle [21–22]. Cyclin A1 is an alternative that is present at very low levels in cells during G0 but it increases throughout the progression of the cell cycle and reaches its highest levels in the S and G2/M phases [23]. The expression of cyclin A1 was significantly high in EC than in normal tissues [24]. Therefore, the anti-oncogenic role of miR-372 in endometrial carcinoma may be similar to its role in cervical carcinoma through the downregulation of Cyclin A1 and CDK2 expression.

Interestingly, we found another target of miR-372 in endometrial carcinoma, namely RhoC, which is involved in the tumorigenesis and development of many kinds of malignant tumors in humans. According to the 3′UTR of miR-372 and luciferase reporter assays, we found that RhoC is a direct target of miR-372. We confirmed this observation by showing that the expression levels of RhoC in miR-372-transfected cells and in the tumor tissues of the HSA-372 group of nude mice were downregulated after transfection or injection, respectively.

RhoC belongs to the highly conserved Ras homologous subfamily, which plays an important role in tumor cell metastasis and invasion [25]. Upregulated expression of RhoC is related to the metastasis and invasiveness of many kinds of human malignant tumors, such as non-small cell lung carcinoma and hepatocellular carcinoma cells [26–37] and is correlated with a poorer prognosis in patients with pancreatic adenocarcinoma and ovarian carcinoma [38–41]. Furthermore, RhoC promotes the proliferation of gastric cancer cells [42] and inflammatory breast cancer (IBC) [43] and regulates apoptosis of hepatocellular carcinoma cells [44]. Thus we knocked out RhoC by siRhoC transfection in EC cells and found that their proliferation, migration and invasion ability was suppressed, besides, RhoC downregulation caused G1 phase arrest and promoted cell apoptosis. Consequently, we suggest that the anti-oncogenetic effect of miR-372 in EC may be through targeting the downregulation of RhoC. This led us to determine the expression of relevant genes [45–51], which are involved in the promoting role of RhoC in malignant tumors after transfection with miR-372 in EC cells. We found that the expression levels of MMP2 and MMP9 were consistent with RhoC and contrary to miR-372, but that cleaved PARP and BAX exhibited the opposite trend. Moreover, their expression in the tumor tissues of the HSA-372 group of nude mice showed the same trend. Taken together, these results demonstrate that miR-372 inhibits the tumorigenesis and development of endometrial cancer by targeting RhoC through the regulation of a series of relevant genes.

MicroRNA and their specific targets are dependent on the specific cellular environment [52]. Therefore, the miR-372/RhoC pathway and the miR-372/Cyclin A1 and CDK2 pathway may collectively be involved in the anti-oncogenic properties of miR-372 in endometrial carcinoma. The present research is first to indicate the anti-oncogenetic role of miR-372 in endometrial carcinoma, and it may provide a new insight into the diagnosis and therapy of endometrial carcinoma.

## MATERIALS AND METHODS

### Endometrial carcinoma specimens

63 Endometrial adenocarcinomas (ECs) and 20 normal endometrial specimens were collected from patients who underwent surgical resection at the Department of Gynecology of the First Affiliated Hospital of China Medical University (Shenyang, Liaoning, China). Of the EC cases, 53 underwent lymph node dissection. The tumor specimens were independently confirmed by two pathologists. Samples were frozen immediately in liquid nitrogen and stored at − 80°C until use. None of the patients had preoperative chemotherapy or radiotherapy. Informed consent was obtained from all subjects, the study was approved by the China Medical University Ethics Committee, and all specimens were handled and made anonymous according to ethical and legal standards.

### Cell culture and transfection

The human endometrial carcinoma cell lines HEC-1B and Ishikawa were obtained from the Tumor Cell Bank of the Chinese Academy of Medical Science (Peking, China). The HEC-1B cells were cultured in Modified Eagle's Medium (DMEM; HyClone, Logan, UT, USA) and Ishikawa cells were cultured in RPMI 1640 (HyClone, Logan, UT, USA) supplemented with penicillin/streptomycin (100 U/ml) with 10% fetal bovine serum (FBS) under conditions of 5% CO_2_ at 37°C. All transfections were carried out using Lipofectamine 2000 according to the manufacturer's instructions. The concentration of miR-372 (Biomics Biotech, Jiangsu, China) was 50 nM. The sequence of miR-372 mimic was 5′-AAAGUGCUGCGACAUUUGAGC GUGCUCAAAUGUCGCAGCACUUUUU-3′. The target sequences of RhoC siRNA were 5′-GUGCCUUUGG CUACCUUGAdTdT-3′ (sense) and 5′-UCAAGGUA GCCA AAGGCACdTdT-3′ (anti-sense).

### MTT assay

The cells were seeded in 96-well plates at a density of 3,000 cells/well for different periods. At a given time point (0 h, 24 h, 48 h, and 72 h) after transient transfection, the cells were incubated with 20 μl of 5 mg/ml MTT (Solarbio, Beijing, China) at 37°C for another 4 h. Then the medium was removed, and the precipitated formazan was dissolved in l50 μl of DMSO. After shaking for 10 min, the absorbance at 490 nm was detected using a microplate spectrophotometer (Bio-Tek Instruments, Winooski, VT).

### Cell cycle analysis

The cells were trypsinized, collected, washed, and fixed in 70% ice-cold ethanol for at least 12 h, and then were washed again and resuspended in buffer solution (400 μl) with RNaseA (0.125 mg) at 37°C for 30 min. The cells were stained with propidium iodide (100 μl) (PI; Key Gen, Nanjing, China) and incubated at 4°C in the dark for 30 min. The cell cycle profiles were determined by flow cytometer.

### Apoptosis assay

Briefly, cells were harvested at 2000 r/min for 5 min and washed twice with cold PBS. Then cells were resuspended in 200 μL binding buffer and 5 μL annexin V–FITC at 1 × 10^6^ cells/mL and incubated for 15 min at room temperature in the dark. A total of 300 μL binding buffer and 5 μL PI were added to each tube, and the cell apoptosis rate was determined by flow cytometer within 1 h.

### Wound-healing assay

Cells were cultured to 80% confluence in 6-well culture plates before scratched with a 200 μl pipette tip. After scratching, cells were washed with PBS and cultured in FBS-free medium. Wounds were observed by microscope and photographed at 0/24/48 h. The nude areas were measured using Image J software (National Institutes of Health, Bethesda, MD, USA). The wound healing rate = (Area of original wound − Area of actual wound at different times)/Area of original wound × 100%.

### Invasion assay

Matrigel coated transwell cell culture chambers (BD Bioscience, San Jose, CA, USA) were used for the invasion assay. Filters were coated with 30 μl of basement membrane Matrigel at a dilution of 1:10. Cells (5*10^4^/L) resuspended in 200 μl serum-free medium were layered in the upper compartment of transwell inserts. The bottom chambers contained 600 μl complete medium serving as the chemoattractants. After incubation for 48 h at 37°C, cells on the upper surface of the filter were removed using a cotton swab and invaded cells at the bottom of the upper chamber were fixed with formaldehyde, stained with crystal violet, and counted under an Olympus fluorescence microscope (Tokyo, Japan).

### Real-time RT-PCR

Total RNA was isolated from endometrial carcinoma cell lines and tissues with TRIzol reagent (Takara, Shiga, Japan) and was reverse transcribed to cDNA using an avian myeloblastosis virus transcriptase and random primers (Takara, Shiga, Japan) according to the manufacturer's protocol. Then the cDNA was amplified by real-time quantitative PCR with SYBR Premix Ex Taq ™ II kit (Takara, Shiga, Japan). The expression level of each target gene was normalized to 18s mRNA. The data analysis was calculated according to the sample threshold cycle (Ct) value from three independent experiments. Hairpin-it™ microRNA and U6 snRNA Normalization RT-PCR Quantitation (GenePharma) were used to check mature miR-372.

### Western blotting

The total cell proteins were harvested and lysed in RIPA buffer containing protease inhibitors. 40 μg protein lysates were separated on 10% SDS-polyacrylamide gel and electrotransferred to Hybond membranes (Amersham, Munich, Germany). Fat-free milk (5%) was used to block membranes for 2 h at room temperature. After blocking, primary antibodies, including RhoC (1:500, Santa Cruz Biotechnology, Santa Cruz, CA, USA), MMP2, MMP9, PARP, Bax, CyclinA1, CDK2, ATPase family AAA domain-containing protein2 (ATAD2), large tumor suppressor 2 (Lats2), Cyclin-dependent kinase inhibitor 1A (P21), sequestosome1 (P62), and Dickkopf-1 (DKK1) (1:500, Proteintech, Proteintech Group, USA) were incubated with the blot overnight at 4°C. The next day, the secondary antibody (1:5000) was added for 2 h at room temperature after the membrane was washed three times with TBST. Protein was visualized using an enhanced chemiluminescence (ECL) system according to the manufacturer's protocol (Santa Cruz Biotechnology, Santa Cruz, CA, USA). Anti-GAPDH (1:2000, Proteintech Group, USA) was used as the inner control.

### Immunohistochemistry

Paraffin-embedded tissue sections were deparaffinized in xylene and rehydrated in a graded series of ethanol solutions and then incubated for 20 min in 3% H_2_O_2_ to quench the endogenous peroxidase activity. Next, the sections were heated in target retrieval solution (Dako) for 15 min in a microwave oven (Oriental Rotor) to retrieve the antigen. Non-specific binding was blocked by incubating with 10% goat serum for 2 h at room temperature. The slides were then incubated over night at 4°C with anti-RhoC primary antibodies. Subsequently, an appropriate secondary antibody was added and incubated for 1 h at 37°C, and the binding was visualized with 3, 39-diaminobenzidine tetrahydrochloride (DAB). After each treatment, the slides were washed three times with TBST for 5 min.

### *In vivo* nude mouse xenograft assay

All animal experiments were undertaken in accordance with the National Institutes of Health Guide for the Care and Use of Laboratory Animals with the approval of China Medical University Animal Care and Use Committee. Female BALB/c nude mice, 4–6-weeks-old were obtained from Vital River Laboratories (VRL; Beijing, China) and were routinely housed in light (12 h dark/12 h light) and temperature-controlled rooms. Animals had free access to food and water. HEC-1B [53] cells (1 × 10^7^) were transfected with mutant or wild HSA-372 were resuspended in 200 μL FBS-free culture medium, and injected subcutaneously into the right flanks of mice. The tumor volume and tumor weight was measured routinely following inoculation using direct measurement and calculated using the formula (length × width^2^)/2.

### Dual-luciferase reporter assay

HEK293T cells were co-transfected with either RhoC 3′UTR clone or negative control clone and miR-372 or scramble control using Lipofectamine 2000 reagent 24 h after plating in 24-well plates. PRL-TK vector expressing renilla luciferase was used as the transfection control. Cell extracts were prepared 48 h after transfection, and luciferase activity was measured using the Dual-Luciferase Reporter Assay System (Promega, USA) according to the manufacturer's protocol. The ratio of firefly to renilla luciferase signal was used to normalize firefly activity for intra-experimental transfection efficiency. The wild sequence for RhoC (NM_001042679) 3′ UTR: AGCCACGCCTATGCCCTGCCCTTCCTCAGGGCCCCT GGGGATCTTGCCCCCTTTGACCTTCCCCAAAGGAT GGTCACACACCAGCACUUTTACACTTCTGGCTCAC AGGAAAGTGTCTGCAGTAGGG; while mutant was AGCCACGCCTATGCCCTGCCCTTCCTCAGGGCCCCT GGGGATCTTGCCCCCTTTGACCTTCCCCAAAG GAT GGTCACACACCUAUACGCTTACACTTC TGGCTCA CAGGAAAGTGTCTGCAGTAGGG (Shanghai Genechem Co., Ltd, Shanghai, China).

### Statistical analyses

Statistical analyses were performed using SPSS 17.0 (SPSS, Chicago, IL, USA). The differences among groups, in at least three separate experiments, were analyzed using a double-sided Student's *t*-test. *P* < 0.05 was considered to indicate a statistically significant difference.
